# Markers aiding the diagnosis of chondroid tumors: an immunohistochemical study including osteonectin, bcl-2, cox-2, actin, calponin, D2-40 (podoplanin), mdm-2, CD117 (c-kit), and YKL-40

**DOI:** 10.1111/j.1600-0463.2009.02461.x

**Published:** 2009-07

**Authors:** SØREN DAUGAARD, LISE H CHRISTENSEN, ESTRID HØGDALL

**Affiliations:** 1 Department of Pathology, RigshospitaletCopenhagen, Denmark; 2 Department of Pathology, Bispebjerg HospitalCopenhagen, Denmark; 3 Department of Pathology, Herlev Hospital and Danish Cancer SocietyCopenhagen, Denmark

**Keywords:** Chondrosarcoma, chordoma, chondromyxoid fibroma, chondroblastoma, osteonectin, bcl-2, cox-2, actin, calponin, D2-40 (podoplanin), mdm-2, CD117 (c-kit), YKL-40

## Abstract

Chondroid tumors comprise a heterogenous group of benign to overt malignant neoplasms, which may be difficult to differentiate from one another by histological examination. A group of 43 such tumors was stained with nine relevant antibodies in an attempt to find consistent marker profile(s) for the different subgroups. Archival material from three extraskeletal myxoid chondrosarcomas, five chordomas, five chondromyxoid fibromas, five chondroblastomas and 25 chondrosarcomas was stained with antibodies against osteonectin, bcl-2, cox-2, actin, calponin, D2-40 (podoplanin), mdm-2, CD117 (c-kit) and YKL-40. All 25 chondrosarcomas showed a positive staining reaction for D2-40, none for actin and CD117, and a partial reactivity for bcl-2 (36%). Chondroblastomas (5/5) and chondromyxoid fibromas (2/5) were the only tumors with a positive reaction for actin, and all chondroblastomas (n=5) and extraskeletal myxoid chondrosarcomas (n=3) were positive for bcl-2. In contrast to all other tumors, two of three extraskeletal myxoid chondrosarcomas were also positive for CD17 and negative for osteonectin, cox-2, mdm-2 and actin. All five chordomas were negative for D2-40 and positive for mdm-2 and YKL-40. The diagnosis of chondrosarcoma may be aided by its positivity for D2-40 and YKL-40 and its lack of reactivity for actin and CD117. This should be seen in the light of no reaction for D2-40 in chordomas and a corresponding lack of reaction for osteonectin, cox-2, mdm-2 and actin in extraskeletal myxoid chondrosarcomas. A convincing immunoreactivity for calponin and/or actin in chondromyxoid fibromas and chondroblastomas may also be helpful in differentiating these tumors from chondrosarcomas.

Chondroid tumors are rare, the most common subtype being the *chondrosarcoma*, with an incidence of about two per million per year (1). *Chondromyxoid fibromas*, *chondroblastomas* and *chordomas* are less common, but they are difficult to differentiate from chondrosarcomas because of overlapping histological morphology and limited experience in diagnosing these rare tumors. In contrast to the malignant chondrosarcoma, chondromyxoid fibroma and chondroblastoma are benign, generally indolent lesions, but they may recur and threaten the function of a neighboring joint. The behavior of *chordomas*, on the other hand, is impossible to predict from histology alone. Some tend to recur locally, and some are frankly malignant and metastasize.

An exact subclassification of chondroid tumors by means of markers that reveal the different molecular genetic characteristics is of importance for future therapeutic treatments. Chondrosarcomas and chordomas are resistant to both chemotherapy and radiation, and the mainstay of treatment today is radical surgery.

Finding an antibody panel against markers, which could be of help in the diagnosis of these rare chondroid tumors, thus differentiating benign from potentially malignant subtype, was one goal of this study. The other was finding marker(s) of future therapeutic value. We included D2-40 (podoplanin), a known marker of chondrosarcoma (2), bcl-2, an inhibitor of apoptosis and effector of the Indian hedgehog/parathyroid hormone-like hormone pathway, and COX-2, a mediator of angiogenesis, both molecular markers of possible therapeutic targeting [for a review, see (3)], YKL-40, a ‘high activity’ marker (4, 5), osteonectin (SPARC or BM-40), a known marker of osteosarcomas, chondrocytes and other mesenchymal cells (6), the muscle/myofibroblastic markers actin (7) and calponin (8), mdm-2, a marker of liposarcomas (9) and CD117 (c-kit), a marker of gastrointestinal stromal tumors with mutations in c-kit and PDGFRα (10).

The markers were applied by immunohistochemistry to a series of 43 chondroid tumors of the categories mentioned above. For comparison, three extraskeletal myxoid chondrosarcomas were included, although these tumors, in spite of their name and histologic resemblance to myxoid chondrosarcomas (grade II) of bone origin, are considered non-chondroid and have been reclassified as ‘tumors of uncertain differentiation’ in the recent 2002 WHO classification (4).

## MATERIALS AND METHODS

From the archives of the Department of Pathology at Rigshospitalet, we retrieved slides and paraffin blocks from and confirmed the diagnosis of three extraskeletal myxoid chondrosarcomas, five chordomas, five chondromyxoid fibromas, five chondroblastomas and 25 chondrosarcomas of different grades. Because of severe problems with inter-observer variation in the grading of the latter, it was decided to re-use material from a previous study, in which grade had been established through consensus among three experienced observers (11).

To secure adherence of the chondroid tissue to the slides, these were baked dry in an oven at 60 °C for 40 min and then kept at 37 °C for 65 h. The procedure did not affect immunohistochemical staining results, with the exception of the marker bcl-2, which is heat labile. Dilution of this antibody was consequently adjusted to 1:50 as opposed to 1:400, which is used in daily routine.

The antibodies and their dilutions are listed in Table 1. Heat-induced epitope retrieval was performed in a microwave oven (15 min at 98 °C in Tris-EGTA buffer, pH 9). The slides were incubated for 1 h at room temperature and routinely processed with the reactions visualized using Dako's EnVision kit with DAB chromogen on a Dako TechMate staining machine (Glostrup, Denmark).

**Table 1:** List of antibodies

Name	Clone	Supplier	Dilution	Control tissue
Osteonectin/SPARC	ON1-1	Zymed	1:6000	Osteosarcoma
BCL2 oncoprotein	124	Dako	1:50	Lymph node
Cox-2	SP21	Neomarkers	1:800	Intestine
Actin (smooth muscle)	1A4	Dako	1:400	Intestine
Calponin	CALP	Dako	1:300	Intestine
Mdm-2	1F2	Zymed	1:50	Liposarcoma
D2-40, podoplanin, Aggrus	D2-40	Dako	1:50	Intestine
CD117, c-kit	Rabbit polyclonal	Dako	1:200	Intestine
YKL-40	209F9	see “Material and methods”	1:100	Fetal cartilage

Staining for YKL-40 was carried out manually, because the procedure differed from that above: antigen retrieval was performed in a conventional oven (18 h at 60 °C in Tris-EGTA buffer, pH 9). After peroxidase blocking, the sections were pre-incubated with 5% purified bovine serum albumin (BSA) (Dade Behring, Marburg, Germany) for 10 min, and then incubated with primary mouse monoclonal antibody anti-human YKL-40 (IgG2b clone 201 lot no. F9, 3.8 mg/ml, directed against epitopes in amino acid positions 210–220; supplied by Paul A. Price, UCSD, La Jolla, CA, USA) diluted in 1% BSA for 1 h. The reaction was visualized with Dako's EnVision System as above. Apart from the antigen retrieval, all steps were performed at room temperature in a humidity chamber to avoid drying out of the sections.

Staining results were scored semi-quantitatively (0=no staining, +=weak staining, and ++=moderate to intense staining), and the staining pattern was noted (focal/diffuse/varying in intensity). For bcl-2 (cytoplasmic reaction), cox-2 (cytoplasmic reaction) and mdm-2 (nuclear reaction), a percentage was estimated by counting 100 cells, if possible.

## RESULTS

### The staining results are summarized in Table 2

#### Extraskeletal myxoid chondrosarcomas (three cases). –

**Fig. 1:**
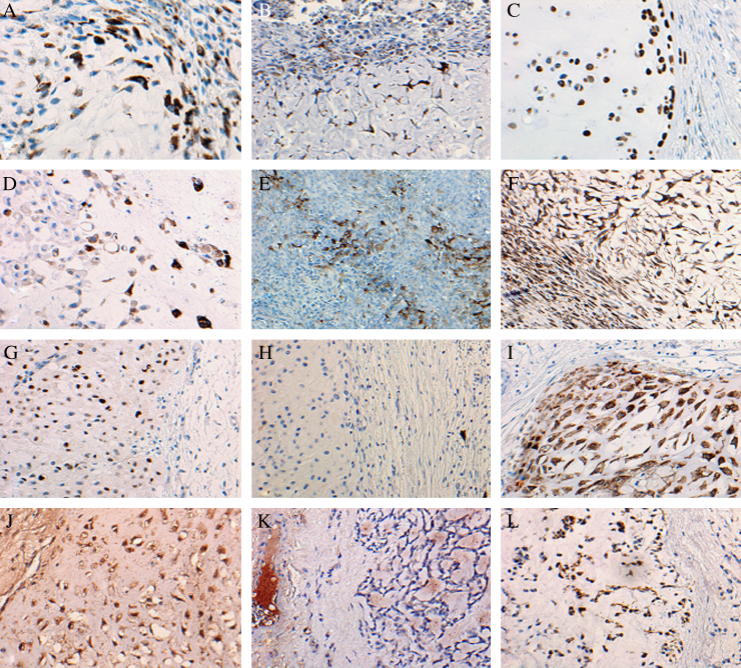
(A) Osteonectin, chondrosarcoma grade III. (B) Osteonectin, chondromyxoid fibroma. (C) Bcl-2, chondrosarcoma grade II. (D) Cox-2, chordoma. (E) Actin, chondroblastoma. (F) Calponin, the single chondromyxoid fibroma with a ++ reaction. (G) Mdm-2, chordoma. (H) D2-40 (podoplanin), advancing edge of chordoma with soft tissue invasion. The only positivity is seen in compressed lymph vessels. (I) D2-40 (podoplanin), chondrosarcoma grade II. (J) YKL-40, chondrosarcoma grade II. (K) YKL-40, ESMC. Note staining of the contents in the blood vessel. (L) CD117, ESMC. All magnifications originally × 200.

Two cases were well differentiated (classical). Both were positive for CD117 (Fig. 1L) and to some degree also for bcl-2. They were only weakly positive for D2-40. The other markers, including YKL-40 (Fig. 1K), were negative. One case was anaplastic, with severe pleomorphism and necrosis: this tumor was negative for CD117, but showed focal positivity for calponin and D2-40 and a variable, weak to moderate staining for YKL-40. A very weak cytoplasmic reaction for cox-2 was seen in some of the tumor cells bordering foci of necrosis, presumably non-specific.

**Table 2:** Summary of immunohistochemical staining results

Diagnosis	No.	Osteonectin	bcl-2	cox-2	mdm-2	D2-40	YKL-40	Actin	Calponin	CD117
Extraskeletal myxoid chondrosarcoma	2 conventional	–	2+	–	–	2+	2−	–		2++
	1 anaplastic	–	1++	–	–	1+	1+ (var)	–	1+	1−
Chordoma	5	3−	–	3−	5+	–	3+	–	1+	–
		2+		2+	(4–45%)		2++	–		–
				(10–40%)						
Chondromyxoid fibroma	5	2−	5+	2−	2−	2−	5+	3−	4−	–
		3+ (var)	(5–50%)	3+ (50%)	3+ (var)	3+ (var)		2+	1++	
Chondroblastoma	5	3−	–	5+	3−	3+	2−	5+	3+	–
		2+		(5–50%)	2+	2++	3+ (focal)			
					(1–2%)					
Chondrosarcoma	10 grade 1	2−	8−	8−	7−	10++	8+	–	4 (weak)	–
		8+ (var)	2+	2+ (var)	3+		2++			
					(10–40%)					
	12 grade II	11+	6−	3−	7−	2+	7+	–	–	–
		1++	6+/++	9+ (var)	5+	10++	5++			
					(2–10%)					
	3 grade III	1−	2−	1−	–	3++	2+	–	–	–
		1+	1+	2+			1++			
		1++								

#### Chordomas (five cases). –

Two cases exhibited a weak positivity for osteonectin, mainly peripherally in the tumor nodules. No reaction was seen for bcl-2, actin, D2-40 (Fig. 1H) or CD117, and only one case exhibited a weak positivity for calponin. Two cases were positive for cox-2 (about 10% and 40% of tumor cells, respectively; Fig. 1D). All showed some reactivity for mdm-2 (4–45% of nuclei) (Fig. 1G) and YKL-40, which, however, was highly variable. One case, with only 4% mdm-2-positive cells and a weak (+) reaction for YKL-40, appeared morphologically to have been suboptimally fixed.

#### Chondromyxoid fibromas (five cases). –

The chondroid and myxoid areas were negative for osteonectin, except in one case, where about 25% of the tumor cells were positive (Fig. 1B). Otherwise, areas with reactive bone formation and scattered cells in the spindled areas exhibited some positivity (possibly myofibroblasts). Cox-2 stained about 50% of the myxochondroid tumor cells in three cases, whereas two were negative. Bcl-2 exhibited a generally weak and focal positivity in three cases (5–10% of tumor cells), while two cases were more strongly positive (20–50% of tumor cells). Of the myogenic markers, actin was focally positive in only two cases and calponin was strongly positive in one (Fig. 1F). Mdm-2 exhibited a varying nuclear positivity in three cases (from 5% to 35%), and the same three cases were also positive for D2-40. CD117 was negative, and YKL-40 was positive in the chondroid areas in all cases, albeit with varying intensity.

#### Chondroblastomas (five cases). –

Osteonectin was invariably negative in the cellular areas and centrally in the obvious chondroid areas, but in two cases positivity was encountered in myxoid spindle cell areas and peripherally in the chondroid. Cox-2 was positive in all cases (varying between 5% and 50% of tumor cells). Also, all cases exhibited at least focal positivity for actin (Fig. 1E), and three for calponin. No significant reaction was seen for mdm-2: only two cases exhibited a weak positivity in 1–2% of tumor cells. No reaction was seen for bcl-2 and CD117. D2-40 was positive in all cases but with varying intensity. YKL-40 was negative in two cases and positive in three – only in the chondroid areas.

#### Chondrosarcomas (25 cases). –

A variable and often focal positivity was seen for osteonectin – in some cases most intense in the periphery of the tumor nodules (Fig. 1A). Bcl-2 exhibited variable positivity in nine cases, six of them being grade II (Fig. 1C). Cox-2 was negative in 12 cases, while 13 showed a varying positivity, ranging from 4% to 50%, with a considerable intra-tumoral variation. Actin was negative in all cases. Calponin was also negative, apart from four cases exhibiting a doubtful, weak, focal positivity. Eight cases exhibited nuclear positivity for mdm-2, varying between 2 and 40% of nuclei. A cytoplasmic reaction was present in a larger proportion of tumor cells in the same cases, and one case showed only cytoplasmic staining (not regarded as positive). A generally moderate or strong reaction was seen for both D2-40 and YKL-40 (Fig. 1I and J). No reaction was seen for CD117.

## DISCUSSION

The diagnosis of chondroid tumors and their mimics can be difficult to make outside large referral centers, and even here, where it is usually accomplished by a combination of microscopy and clinical findings (including image diagnostics), difficulties may occur, if the material is sparse, poorly preserved or traumatized. The results of this study are therefore encouraging, as some of the antibodies appear to be of assistance in subclassification of the tumors, and some of the markers may even point to new therapeutic strategies in the future.

The most significant finding was the universal immunoreactivity of the 25 chondrosarcomas for both D2-40 and YKL-40. Both of these failed to show any consistent patterns for the other tumors, and as D2-40 was completely negative in all our chordomas – an observation confirmed by Huse et al. (12) and to some extent also Oakley et al. (2) – it may be of help in distinguishing these tumors from chondrosarcomas. The transcription factor brachyury, a regulator of notochordal development, has recently proven highly specific as a positive marker of chordomas as opposed to other chondroid tumors (2, 13), and these two markers are likely to effectively supplement each other immunohistochemically in the distinction between chordomas and chondrosarcomas.

D2-40, an antibody with several names (podoplanin, Aggrus, M2A and hT1α-2) is directed against a transmembrane glycoprotein, and its main use in diagnostic pathology is as a marker of lymphatic endothelium, germinal cell tumors and mesotheliomas, but it also reacts with myofibroblasts, chondrocytes and osteocytes (2, 14). The antibody may also function as a prognostic marker. Experimental evidence has shown that over-expression promotes metastasis, which may lead to a more aggressive therapeutic intervention (15).

The essential driving force for cartilage differentiation is the Indian hedgehog/parathyroid hormone-like hormone pathway. Studies have suggested that the transition from osteochondroma to chondrosarcoma is characterized by reactivation of the Indian hedgehog/parathyroid hormone-like hormone signalling, and that bcl-2, an effector of this, is expressed in peripheral and high-grade central chondrosarcomas [for a review, see (3)]. Our results regarding bcl-2 should probably be interpreted with caution. The antigen is heat labile and even though the concentration of the antibody had to be increased the lymphocytic reaction was in some cases extremely weak, presumably due to over-decalcification. However, in spite of these practical shortcomings, we found positivity in two of 10 grade I, six of 12 grade II and two of three grade III chondrosarcomas, in accordance with the findings of Bovée et al.(16) and Hameetman et al.(17), that up-regulation of bcl-2 is an early event in some (peripheral) tumors and a late occurrence in others (central ones). Unfortunately, it has not been possible for us to classify the tumors reliably as either peripheral or central in this retrospective material. Bcl-2 reactivity was also seen in the chondromyxoid fibromas while the chondroblastomas were negative, indicating different paths of tumorogenesis for these two entities despite their superficial resemblance.

*Extraskeletal myxoid chondrosarcomas* are not known to have a diagnostic immunohistochemical profile (18), but our two classic cases distinguished themselves from the other entities in being positive for CD117 and at least focally for bcl-2, while they were mainly negative for YKL-40 (one case exhibited a weak, granular reaction). CD117 (c-kit) positivity has been reported previously. Hornick and Fletcher (19) found positivity in two of 20 tumors, and Subranian et al. (20) in six of 10, but the latter group did not find any mutations in the *KIT* gene, indicating that treatment with the tyrosine kinase inhibitor imatinib is unlikely to have any effect. Yet, the marker may be helpful in the differential diagnosis, as all the other tumors were negative for CD117. The mainly negative reaction for YKL-40 is somewhat surprising, because Sjögren et al.(21), in their 10 cases, demonstrated over-expression of the *CHI3L1* gene (which codes for YKL-40) by RT-PCR. However, they did not perform immunohistochemical staining for the protein. Positivity for bcl-2 has, to our knowledge, not been described before, but it should be worth investigating further, especially because the over-expression of this apoptosis-preventing protein is the target of several new strategies of therapeutic intervention (22). Also, silencing of the gene with small interfering RNA has recently been shown to enhance radio-sensitivity *in vitro* (23). The single poorly differentiated extraskeletal myxoid chondrosarcoma in our material did not exhibit the same profile. This might be due to dedifferentiation, but another explanation could be that this subtype is not truly related to the more differentiated, classical ones.

At least focal reactivity for smooth muscle actin was found in all chondroblastomas and a weak positivity was seen in two of the five chondromyxoid fibromas. In both entities, actin positivity is interpreted as signifying myofibroblastic differentiation (7, 8), which, in the chondromyxoid fibromas, is driven by the TGF-β1 pathway (8). The focal and weak reaction for osteonectin also occurred primarily in myofibroblast-like cells. Both benign tumors exhibited a varying positivity for D2-40 and YKL-40, mainly in areas with visible chondroid differentiation. One of the chondromyxoid fibromas was strongly positive for calponin. This is in contrast to the results of Romeo *et al.* (8), who found no reaction for calponin in their 20 cases.

Osteonectin (also known as SPARC or BM-40) is a secreted Ca-binding glycoprotein that plays a role in the development of mesenchymal cells, especially during the transformation of chondroid tissue into bone, and recent interest in this protein centers upon its role in response to injury and tumor progression (24). Immunohistochemically, osteonectin is known to stain osteosarcomas, but it also decorates chondrocytes, fibroblasts and other non-neoplastic cell types (6). The reaction pattern for osteonectin was not informative, except for its absence in the extraskeletal myxoid chondrosarcomas and its tendency to be more intensely positive toward the periphery of the chondroid nodules of chondrosarcomas and chondromyxoid fibromas, possibly reflecting the enchondral ossification process.

Mdm-2 has been reported to be a useful marker in liposarcomas (9), but is less well studied in bone tumors. The p53/mdm2 pathway appears to be involved in 40–50% of chondrosarcomas and correlated to aggressive behavior (25) but there is no agreement on the cut-off level for positivity, and we did not find any correlation to histological grade, where eight of 25 (32%) exhibited nuclear positivity for mdm-2 in 2–40% of tumor cells.

In accordance with others (26), we did not find any expression of CD117 (c-kit) in any tumors apart from the extraskeletal myxoid chondrosarcomas. The reported effect of imatinib in the latter group may be mediated through auto- or paracrine activation of PDGFRβ, or other pathways (10).

YKL-40, also known as human cartilage protein 39 or *CHI3L1*, is a secreted glycoprotein whose precise function is unknown. It occurs ubiquitously in normal adult human tissues, and the staining intensity correlates with cellular activity (metabolic or proliferative) (4, 26). During fetal development, it acts as a growth factor for chondrocytes and fibroblasts, with differential expression during the formation of bones and joints (26), but YKL-40 is also expressed in some cancers, and although no direct correlation between YKL-40 expression in tumor tissue and corresponding serum/plasma levels has been demonstrated, elevated values of the latter are correlated with a poor prognosis (27). However, it should be kept in mind that elevated YKL-40 serum/plasma levels are also seen in non-malignant diseases, e.g. bacterial infections, osteoarthritis or rheumatoid arthritis (26). This is the first study of YKL-40 expression in chondroid tumors, and as normal chondrocytes stain for this marker (4), it is hardly surprising that it was also found in the chondroid tumors. The two classical extraskeletal myxoid chondrosarcomas in this study were negative for YKL-40, and the anaplastic one was only weakly positive. These tumors are probably not histogenetically related to true chondrosarcomas. Gene profiling has indicated neural/neuroendocrine differentiation (20, 21), and expression of YKL-40/*CHI3L1* has been demonstrated by RT-PCR in 10 cases of extraskeletal myxoid chondrosarcomas, but without performing immunohistochemical staining for the protein (20), and the discrepancy may be caused by posttranslational modification. As a routine immunohistochemical marker, YKL-40 is not optimal, because it is too widespread, but it may be of value in the postoperative follow-up of patients with potentially malignant chondroid tumors, and in the monitoring of patients with multiple osteochondromas or enchondromas, who have an increased risk of developing chondrosarcomas.
